# Effect of Angiogenesis and Lymphangiogenesis in Diesel Exhaust Particles Inhalation in Mouse Model of LPS Induced Acute Otitis Media

**DOI:** 10.3389/fcimb.2022.824575

**Published:** 2022-05-11

**Authors:** Byeong-Gon Kim, Da Yeon Choi, Min-Gyoung Kim, An-Soo Jang, Myung-Whan Suh, Jun Ho Lee, Seung Ha Oh, Moo Kyun Park

**Affiliations:** ^1^ Department of Otorhinolaryngology-Head and Neck Surgery, Seoul National University College of Medicine, Seoul, South Korea; ^2^ Sensory Organ Research Institute, Seoul National University Medical Research Center, Seoul, South Korea; ^3^ Department of Internal Medicine, Soonchunhyang University Bucheon Hospital, Bucheon, South Korea

**Keywords:** diesel exhaust particles, lipopolysaccharides, otitis media, lymphangiogenesis, angiogenesis

## Abstract

Lymphangiogenesis and angiogenesis might have significant involvement in the pathogenesis of otitis media with effusion. This study investigated the effect of diesel exhaust particles (DEP) on inflammation and lymphangiogenesis in a mouse model of acute otitis media (AOM). BALB/c mice were injected with LPS and exposed to 100 µg/m^3^ DEP. The mice were divided into four groups: control (no stimulation), AOM, AOM + DEP, and DEP + AOM.

The effects of DEP inhalation pre- and post-DEP induction were estimated based on measurements of the auditory brainstem response, mRNA levels of lymphangiogenesis-related genes and cytokines, and histology of the middle ear. Cell viability of human middle ear epithelial cells decreased in a dose-response manner at 24 and 48 hours post-DEP exposure. DEP alone did not induce AOM. AOM-induced mice with pre- or post-DEP exposure showed thickened middle ear mucosa and increased expression of TNF-α and IL1-β mRNA levels compared to the control group, but increased serum IL-1β levels were not found in the AOM + Post DEP. The mRNA expression of TLR4, VEGFA, VEGFAC, and VEGFR3 was increased by pre-AOM DEP exposure.

The expression of VEFGA protein was stronger in the AOM + Post DEP group than in any other group. The expression of CD31 and CD45 markers in the mouse middle ear tissue was higher in the Pre DEP + AOM group than in the AOM group. This result implies that pre-exposure to DEP more strongly increases inflammation and lymphangiogenesis in a mouse model of acute otitis media.

## Introduction

Otitis media (OM) is one of the most common infections in young children. It is a common cause of physician visits and hearing loss in children (Rovers, 2008; Monasta et al., 2012). For those under 18 years of age, OM ranked the fifth highest in terms of direct medical spending in the United States. The costs related to OM are estimated at about $3.2 billion (Soni, 2014). OM disturbs the development of language and speech in children, and negatively affects the central auditory nervous system and auditory processing (Colella-Santos et al., 2019). In addition, it induces sleep disturbances, loss of appetite, and behavioral problems. OM deteriorates the quality of life of children and parents (Rovers, 2008; Homøe et al., 2020), and OM-related hearing loss is associated with poorer early academic achievement (Su et al., 2020).

Several studies have suggested that traffic-related air pollution is associated with the development of OM in children (Brauer et al., 2006; Kennedy et al., 2018). Kennedy et al. reported that exposure to air pollution from motor vehicles during the first year of life is associated with OM, bronchiolitis, and pneumonia (Kennedy et al., 2018). Brauer et al. showed that exposure to higher levels of PM2.5, elemental carbon, and NO_2_, as traffic-related air pollutants, were associated with the development of OM in children younger than 2 years old (Brauer et al., 2006). Diesel exhaust particles (DEP) are considered to be the major component of traffic-related air pollution. A meta-analysis showed that the incidence of OM is correlated with higher levels of exposure to particulate matter (PM), which has similar characteristics to those of DEP. The correlation was strong, especially for younger children, and a stronger relationship was found for PM2.5 than for PM10 (Lee et al., 2020). *In vivo* and *in vitro* studies have demonstrated that DEP could contribute to the development of OM. DEP decreased cell viability in human middle ear epithelial cell lines and increased the inflammatory cytokines and mucin gene expression (Song et al., 2012). Growing evidence indicates that DEP is associated with the development of OM. However, when and how DEP exposure affects the middle ear has yet to be clarified.

It is known that DEP exposure induces angiogenesis. Xu et al. reported that DEP exposure significantly increased the expression of vascular endothelial growth factor (VEFG) and hypoxia-inducible factor (HIF)-1 alpha, while decreasing prolylhydroxylase (PHD) 2 expression. DEP exposure increased the vessel volume, blood flow, capillary tube formation, and sprouting *in vivo* and *in vitro* (Xu et al., 2009). Lymphangiogenesis, which refers to the process of lymphatic vessel formation from existing lymph vessels, has a significant role in cancer metastasis, organ graft rejection, and lymphedema (Yamakawa et al., 2018). Angiogenesis is an essential process of organ growth and repair that constitutes an important therapeutic target for cancer, cardiovascular disease, macular generation, and wound healing (Ferrara and Kerbel, 2005).

Lymphangiogenesis and angiogenesis play a significant role in the pathogenesis of OM with effusion (OME) (London and Gurgel, 2014). Jung et al. first detected VEGF in the middle ear fluid of OME patients and middle ear mucosa of chronic otitis media patients (Jung et al., 1999). VEGFR inhibitors have been found to moderate angiogenesis and lymphangiogenesis in the inflamed middle ear mucosa and improve OM (Cheeseman et al., 2011). Our previous study showed that micro-particles increased VEGFA expression in human middle epithelial cells, as increased VEGFA was detected in transcriptome analysis and validated by quantitative real-time polymerase chain reaction (qRT-PCR) (Song et al., 2013). Li et al. reported that VEGF plays a role in the acute phase of OME. The expression of HIF-1α mRNA was found to be correlated with the expression of VEGF and VEGFR-1 mRNA in an LPS-induced OME model (Li and Ye, 2021). It has been reported that the levels of VEGFA and TGF-β cytokines in adenoids with exudative otitis media were higher than in conditions of adenoid hypertrophy alone, prompting the suggestion that VEGFA and TGF-β could be used as additional and objective tests to confirm the clinical diagnosis of OME caused by a bacterial pathogen (Zelazowska-Rutkowska et al., 2020). In addition to its role in OM, VEGF plays an essential role in cochlear function and hearing, the latter of which is exemplified by its association with sensorineural hearing loss (London and Gurgel, 2014). However, the mechanism of DEP exposure-induced lymphangiogenesis and angiogenesis in OM has yet to be well defined. Therefore, the aim of this study was to investigate the effect of DEP on inflammation and lymphangiogenesis in a mouse model of acute OM (AOM). In addition, we exposed mice to DEP pre- and post-infection to investigate the preconditioning effect. To our best knowledge, this is the first study to compare the preconditioning effect of DEP in OM.

## Results

### Exposure of HMEECs to DEP Resulted in Dose-Dependent Reductions in Cell Viability

**Figure 1:**
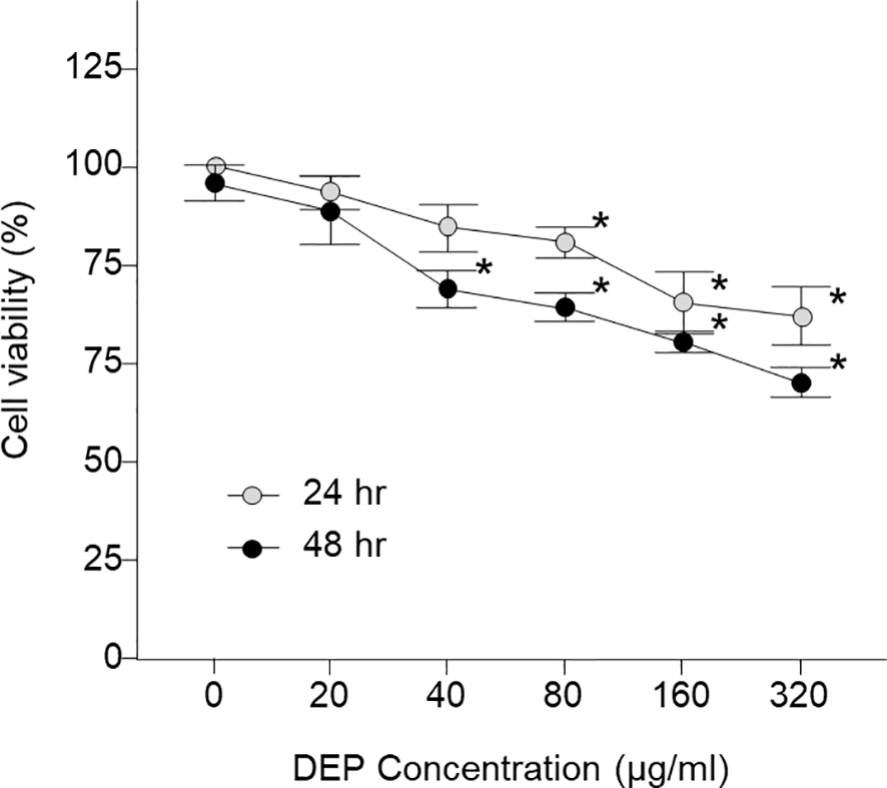
Viability of LPS- and DEP-exposed HMEECs assessed by the CCK-8 assay and study design of the *in vitro* model. LPS and DEP exposure decreased cell viability in HMEECs. *p < 0.05 compared with the 24 hr and 48 hr normal control groups. LPS, lipopolysaccharide; DEP, diesel exhaust particles; HMEECs, human middle ear epithelial cells.

**Figure 2:**
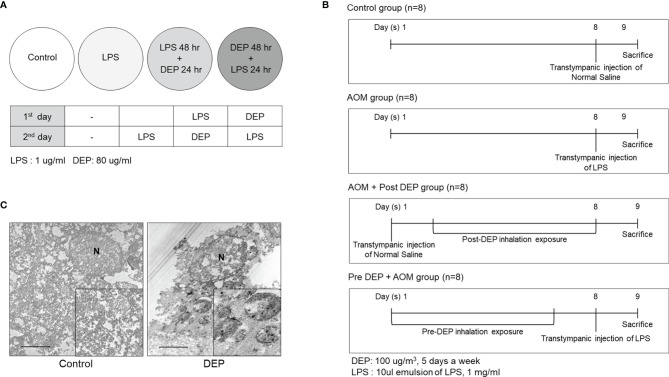
Study design and DEP inhalation in a mouse model of acute otitis media induced by transtympanic LPS injection. **(A)** HMEECs were treated with a cytotoxic dose of LPS (1 µg/mL) and DEP (80 µg/mL) for 24 hr and 48 hr, or both in different sequences or simultaneously as indicated. **(B)** Experimental protocol for DEP exposure in a mouse model of AOM (n = 13 in each group) (DEP 100 µg/m^3^; 10 µL emulsion of 1 mg/mL LPS). **(C)** The DEP were observed in transmission electron microscopy images inside eustachian tube cells connected to tissues only among mice that inhaled 100 µg/m^3^ DEP for 1 hr a day for 5 days a week (N, nucleus; scale bar, 1 µm; magnification ×30,000). LPS, lipopolysaccharide; DEP, diesel exhaust particles; HMEECs, human middle ear epithelial cells; AOM, acute otitis media.

**Figure 1** shows the results when various concentrations of DEP (0, 20, 40, 80, 160, and 320 µg/mL) were added to HMEECs for 24 hr and 48 hr, and cell viability was estimated using the CCK-8 assay; When HMEECs were exposed to DEP, there was no significant difference in cell viability after 24 hr at 40 µg/mL, but a significant difference was found after DEP exposure for 24 and 48 hr at 80 µg/mL. Moreover, as time passed, cell viability significantly decreased compared to the control (both p < 0.05, **Figure 1**). The results showed that DEP induced gradual decreases in cell viability in a dose- and time-dependent manner. Thus, HMEECs were stimulated with a final DEP concentration of 80 μg/mL (**Figures 1**, **2A**).

### Effects of DEP Inhalation on the Severity of Symptoms in a Mouse Model of AOM

**Figure 3:**
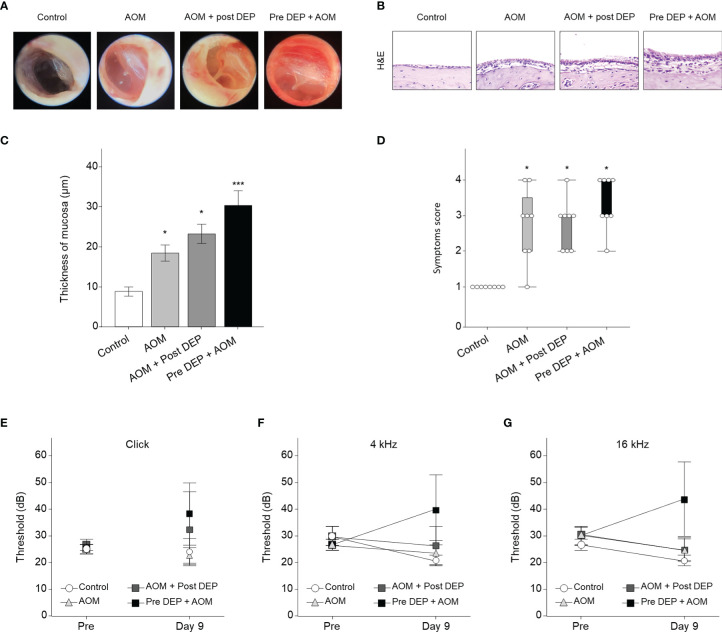
Acute otitis media symptoms and H&E staining analysis of the middle ear in LPS + DEP exposure mouse. **(A)** Otoscope images of the tympanic membrane before sacrifice. **(B)** H&E staining of middle ear pathology. **(C)** The thickness of the middle ear epithelium is higher in the DEP + LPS exposure group. **(D)** The symptom score is higher in the acute otitis media group. **(E–G)** Auditory brainstem response threshold changes in the mouse model. The same location shown in each slide was chosen (H&E, magnification ×400). *p < 0.05, compared with the control group; ***p < 0.05 compared to the AOM group. LPS, lipopolysaccharide; DEP, diesel exhaust particles; H&E, hematoxylin and eosin; AOM, acute otitis media.

The severity of symptoms in the mouse model of AOM was measured the day after the last exposure to DEP (**Figure 2B**). Histopathological examinations revealed typical pathological features of AOM in LPS-induced mice, and the DEP-exposed group exhibited more significant symptoms than the control mice. As shown in **Figure 3**, DEP exposure induced pathologic changes of the tympanic membrane, as assessed by otoscopy. Furthermore, the observed TEM images revealed the presence of particles inside eustachian tube cells connected to middle ear tissues (**Figure 2C**). The tympanic membranes of mice in the control group showed clear structures, but this was not the case for the LPS- and DEP-treated groups (**Figure 3A**). The location shown in the mucosa in **Figure 3B**, near the eustachian tube and in a similar position in each group, was used to measure the mucosa thickness of the middle ear. The thickness of the mucosa significantly increased in the LPS- and DEP-treated groups compared to the control group. In particular, the thickness of the mucosa increased significantly in the Pre-DEP + AOM group relative to the AOM group (p < 0.05, **Figures 3B, C**). The LPS- and DEP-exposed mice exhibited a significant increase in the symptom score and had effusion and changes in the tympanic membrane structure compared with the control group. The effusion and structural changes of the tympanic membrane were exacerbated in the Pre-DEP + AOM group, but the symptom score showed no significant increase in the AOM group (p < 0.05, **Figure 3D**). Hearing in the LPS- and DEP-treated mice was evaluated by auditory brainstem response (ABR) threshold analysis. Before exposure, the Pre-DEP + AOM group showed that the ABR thresholds for click, 4 kHz, and 16 kHz increased after exposure. However, the increases were not statistically significant (**Figures 3E** **–G**). The average hearing levels in the control, AOM, and AOM + Post-DEP groups showed virtually no change, while a non-significant increase was observed in the Pre-DEP + AOM group (**Figures 3E–G**). The LPS- and DEP-exposed mice exhibited a higher symptom score than the control group, and this was exacerbated in the Pre-DEP + AOM group, but the differences were not significant. These results provide support for the possibility that DEP inhalation exacerbates signs and symptoms.

### DEP Triggered IL-1β and TNF-α mRNA Expression in the Mouse Middle Ear and HMEECs

**Figure 4:**
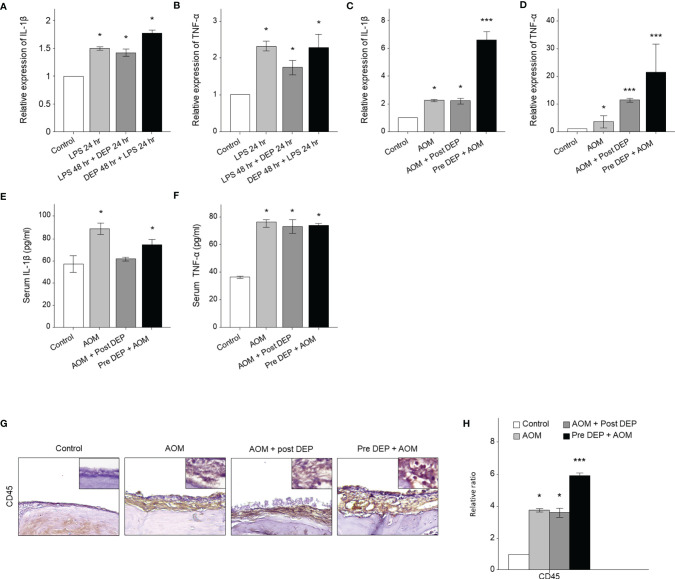
Analysis of cytokine expression in LPS + DEP-exposed mice and HMEECs. The qRT-PCR analysis of mRNA levels of **(A)** IL-1β and **(B)** TNF-α in LPS in HMEECs. The mRNA levels of **(C)** IL-1β and **(D)** TNF-α a mouse model of acute otitis media. The cytokines in the serum of mice were assessed *via* ELISA kits, including **(E)** IL-1β, **(F)** TNF-α. **(G)** Immunohistochemical staining of mouse middle ear tissue using antibodies against CD45 (Microvessel; black arrow, magnification ×400). **(H)** Quantitation of the CD45 staining intensity. *p < 0.05, compared with the control group; ***p < 0.05 compared to the LPS 24 hr and AOM group. LPS, lipopolysaccharide; DEP, diesel exhaust particles; HMEECs, human middle ear epithelial cells; AOM, acute otitis media.

Cytokines play a key role in the pathogenesis of AOM-related mRNA expression of IL-1β and TNF-α in HMEECs and the mouse middle ear. The levels of mRNA encoding IL-1β and TNF-α changed following DEP treatment of HMEECs and the mouse middle ear, as assessed by qRT-PCR. The LPS-exposed groups exhibited greater IL-1β and TNF-α mRNA expression than the control group, and the expression of these cytokines was also higher in the DEP 48 hr + LPS 24 hr group and the Pre-DEP + AOM group than in the LPS 48 hr + DEP 24 hr and AOM + Post-DEP group (p<0.05, **Figures 4A–F**). Although the Pre-DEP + AOM group in the *in vivo* component of the present study showed significantly higher cytokine mRNA levels than the AOM group, no significant differences were found in comparisons with the LPS 24 hr group in the *in vitro* component of the study (**Figures 4A–D**). Serum levels of IL-1β and TNF-α changed following DEP treatment in mice, as assessed by ELISA. The LPS and DEP-exposed group exhibited higher IL-1β and TNF-α mRNA levels than the control group (**Figures 4E, F**), but increased serum IL-1β levels were not found in the AOM + Post-DEP group (**Figure 4E**). The immunohistochemistry results showed that CD45, a marker of immune cell infiltration, increased in the middle ear (**Figures 4G, H**).

### Effect of DEP Inhalation on Angiogenesis and Lymphangiogenesis Signaling Pathways in the Mouse Middle Ear and HMEECs

**Figure 5:**
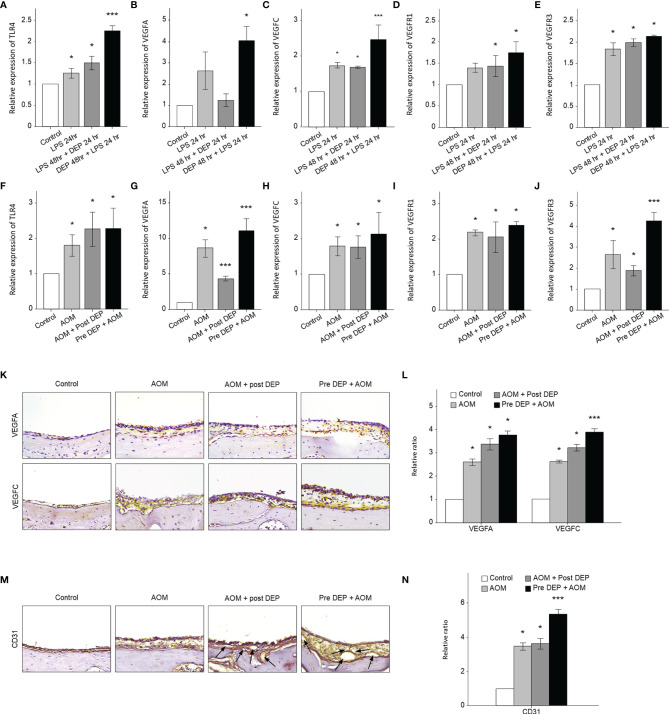
Analysis of the expression of angiogenesis and lymphangiogenesis-related genes in LPS + DEP-exposed mice and HMEECs. The results show the mRNA levels of **(A)** TLR4 and **(B)** VEGFA, **(C)** VEGFC, **(D)** VEGFR1, and **(E)** VEGFR3 in LPS-exposed HMEECs. The mRNA levels of **(F)** TLR4 and **(G)** VEGFA, **(H)** VEGFC, **(I)** VEGFR1, **(J)** VEGFR3 in LPS in a mouse model of AOM. Immunohistochemical (IHC) staining of mouse middle ear tissue using antibodies against **(K)** VEGFA, VEGFC, and **(M)** CD31 (IHC, magnification ×400). The same location shown in each slide was chosen (IHC, magnification ×400). **(L, N)** Quantitation of the CD45 staining intensity. *p < 0.05, compared with the control group; ***p < 0.05 compared to the LPS 24 hr and AOM group. LPS, lipopolysaccharide; DEP, diesel exhaust particles; HMEECs, human middle ear epithelial cells; AOM, acute otitis media.

The mRNA levels of angiogenesis- and lymphangiogenesis-related factors in the mouse middle ear and HMEECs were evaluated by qRT-PCR and immunohistochemistry (**Figure 5**). In DEP 48 hr + LPS 24 hr group in the *in vitro* component of the study and in the Pre-DEP + AOM group in the *in vivo* component of the study, TRLR4, VEGFA, VEGFC, VEGFR1, and VEGFR3 mRNA levels were higher in the control group. However, VEGFA mRNA levels were not significantly increased in the LPS 24 hr, LPS 48 hr + DEP 24 hr groups. Significantly higher TLR4 and VEGFC mRNA levels were found in the DEP 48 hr + LPS 24 hr group than in the LPS 24 hr group (p < 0.05, **Figures 5A, C**). Moreover, VEGFA and VEGFR3 mRNA levels were significantly higher in the Pre-DEP + AOM group than in the AOM group (p < 0.05, **Figures 5G, J**). The expression of the angiogenesis and lymphangiogenesis markers VEGFA and VEGFC in the mouse middle ear tissue was significantly higher in the AOM and DEP-treated groups than in the control group (**Figures 5K, L**). The AOM + Post-DEP and Pre-DEP + AOM groups showed higher VEGFA expression than the AOM group. The expression of CD31, a marker of lymphangiogenesis and angiogenesis marker, in the mouse middle ear tissue was higher in the AOM and DEP-treated groups than in the control group (**Figures 5M, N**). The Pre-DEP + AOM group showed higher CD31 expression than the AOM group, showing that CD31 expression was augmented by DEP treatment.

## Discussion

**Figure 6:**
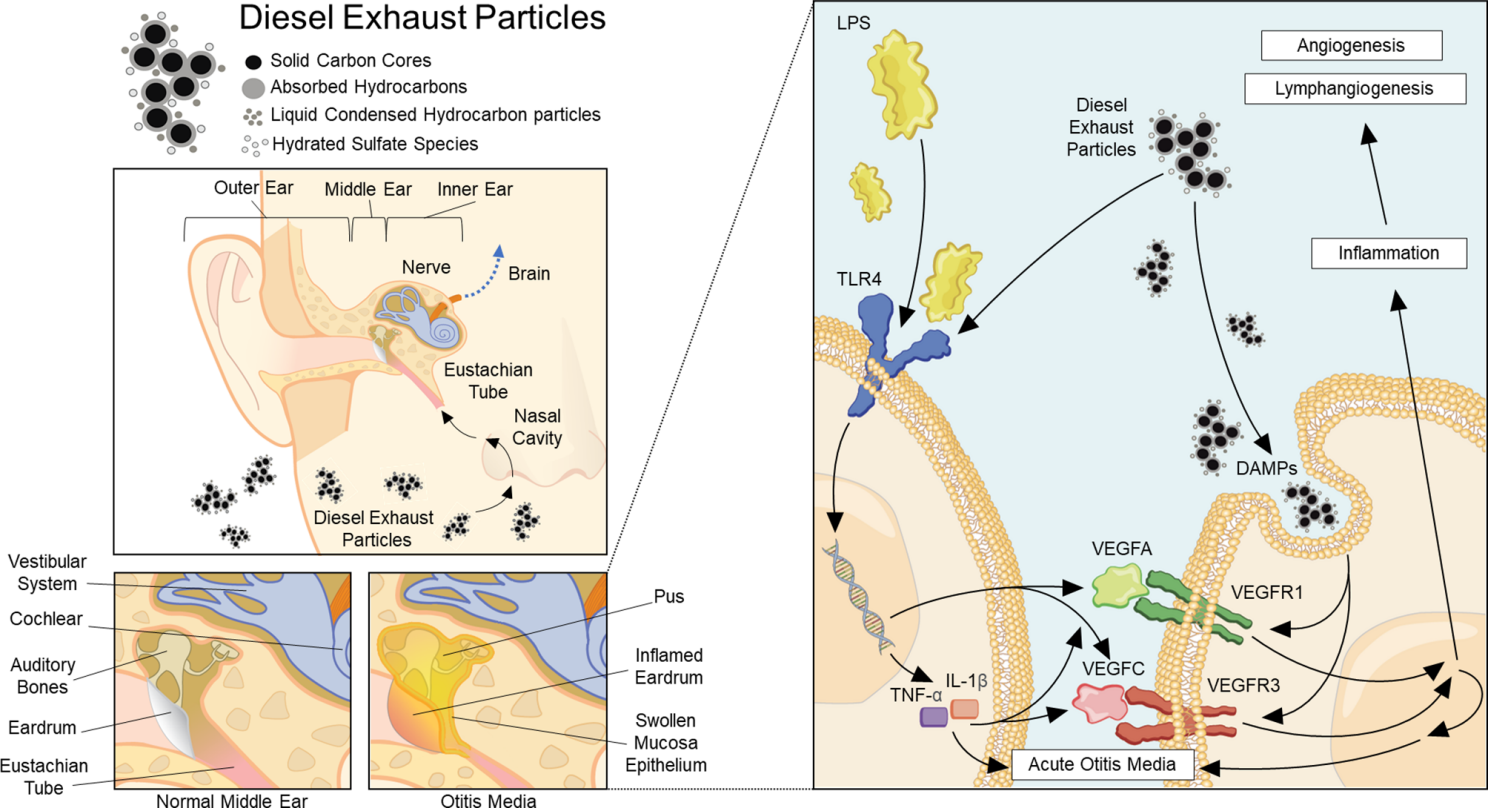
Summary of the pathway of diesel exhaust particle-induced angiogenesis and lymphangiogenesis in acute otitis media.

This study demonstrated that DEP increased VEGF expression in HMEEC lines and an AOM mouse model with pre- or post-AOM DEP exposure (**Figure 6**). Pre-AOM DEP exposure more strongly aggravated the lymphangiogenesis and angiogenesis processes in otitis media.

Li et al. reported that VEGF plays a role in the acute phase of OME. The expression of HIF-1α mRNA was found to be correlated with the expression of VEGF and VEGFR-1 mRNA in an LPS-induced OME model (Li and Ye, 2021). It has been reported that the levels of VEGFA and TGF-β cytokines in adenoids with exudative otitis media were higher than in conditions of adenoid hypertrophy alone, prompting the suggestion that VEGFA and TGF-β could be used as additional and objective tests to confirm the clinical diagnosis of OME caused by a bacterial pathogen (Zelazowska-Rutkowska et al., 2020). In addition to its role in OM, VEGF plays an essential role in cochlear function and hearing, the latter of which is exemplified by its association with sensorineural hearing loss (London and Gurgel, 2014).

In this study, we observed increased vascular formation in the Pre-DEP + AOM group in the animal model and found that LPS increased VEGFA expression both *in vivo* and *in vitro*. Grilli et al. reported that diesel exposure induced the secretion of VEGF and other inflammation biomarkers in transcriptional profiling of human bronchial epithelial cells (Grilli et al., 2018). It is known that VEGF expression is increased after LPS, TNF-α, and IL-1β stimulation (Koyama et al., 2002). Our results demonstrate that VEGF and cytokine levels increased in mice and epithelial cells exposed to DEP or LPS, suggesting that DEP may trigger lymphangiogenesis, angiogenesis, and immune cell infiltration in AOM. The middle ear mucosa has low secretory ability. Vascular leakage is an important process in OM (Rynnel-Dagöö and Agren, 2000). In addition, VEGFR inhibitors moderated angiogenesis and lymphangiogenesis in inflamed middle ear mucosa (Cheeseman et al., 2011). VEGFA, which binds VEGFR1/Flt-1 tyrosine kinase and VEGFR2/Flt-2 tyrosine kinase, is the main inducer of the growth of blood vessels and plays a key role in neurons (Ferrara, 2004). The VEGFA and VEGFC/VEGFR3 pathway is significantly involved in lymphangiogenesis and angiogenesis. Lymphangiogenesis is an important process not only in lymphatic development and disease, but also in cancer treatment and in neurological and cardiovascular diseases (Secker and Harvey, 2021). VEGFR3 is the major receptor for VEGFC. Sweat et al. reported that VEGFC treatment increased blood vessel sprouting and lymphatic sprouting in a rat mesentery culture model (Sweat et al., 2014). It has been reported that VEGFC regulates the proliferation, migration, and survival of lymphatic endothelial cells by VEGFR3 through activating the ERK, AKT, and JNK pathways (Salameh et al., 2005).

Previous studies have shown that DEP and PM decrease cell viability and increase inflammation in the middle ear (Song et al., 2012; Park et al., 2014). We observed the same findings in this experiment. DEP decreased cell viability in a dose- and time-dependent manner in HMEECs. LPS administration, preceded or followed by DEP exposure, induced AOM in a mouse model and showed increased mucosal thickening in the middle ear. Although DEP induced the expression of inflammatory cytokines such as IL-1β and TNF-α *in vivo* and *in vitro*, we did not find a synergistic effect on the expression of TNF-α and Il-1β in the conditions of LPS administration preceded or followed by DEP exposure in HMEECs. Interestingly, however, we found a strong synergistic effect in the Pre-DEP + AOM group in the animal model. The pre-exposed DEP group showed higher levels of inflammatory cytokines and inflammatory cells than the post-DEP exposure group, as well as thicker mucosa. Previous research has shown that PM exerts adverse health effects on the lung and brain, in which fine particles can induce systemic inflammation (Clifford et al., 2018; Kim et al., 2021). However, we did not observe a systemic inflammatory response through inhalation of PM in a mouse model of acute otitis media. A possible explanation for this finding is that LPS may induce the inflammatory response in the middle ear and around the eustachian tube, which may hinder the DEP from reaching the middle ear. In addition, DEP could change the immune response of the middle ear after LPS injection. For instance, it has been reported that macrophage regulation of the inflammatory response to viral infection could be changed by exposure to PM (Becker and Soukup, 1999; Kaan and Hegele, 2003).

In this study, we found different expression patterns of inflammatory cytokines and VEGFs between the *in vivo* and *in vitro* models. These differences may arise from the stimulation time and dose and anatomical characteristics of the middle ear and eustachian tube. *In vitro*, mRNA expression was measured within 24 hr after the last stimulation at 80 µg/mL because cell viability was significantly changed after this point. We investigated the lymphangiogenesis response 1 day after stimulation. *In vivo*, the inflammatory response rapidly increased 1 day after stimulation, peaked around 3 days, and gradually resolved within 14 days (Park et al., 2014). It is known that VEGF and its receptors play a role in the acute phase of OME rather than the chronic or recurrent stage (Ye and Li, 2019). VEGF mRNA expression was detected within 1 hr after instillation, dramatically increased from 6 hours to 1 day, and then progressively decreased by day 7 (Jung et al., 1999).

We used 100 µg/m^3^ DEP for 1 hr a day in the experiment. The 2021 World Health Organization air quality guidelines recommend aiming for annual mean concentrations of PM_10_ not exceeding 15 µg/m^3^ and 24-hour concentrations not exceeding 45 µg/m^3^ (World Health Organization, 2021). A level of 100 µg/m^3^ would be associated with an approximately 2.5% increase in daily mortality (World Health Organization, 2006).

This study showed the increased expression of VEGFC *in vitro*, but not *in vivo*. The mRNA expression of VEGFR3 was higher in the Pre-DEP + AOM group than in the AOM-only or Post-DEP + AOM groups in the animal model. Prior research has established that the expression of Toll-like receptors is increased in AOM, OME, COM, and cholesteatoma in the middle ear. TLR4 recognizes bacterial LPS and endogenous ligands, and induces interleukin expression *via* MyD88 (myeloid differentiation factor 88) and interferon through Toll-like-receptor adaptor molecules (Jung et al., 2021). The expression of TLR2, TLR24, and TLR9 was found to be higher in the middle ear fluid of AOM patients (Kaur et al., 2015). It has been found that DEP increased the expression of mucin genes through the TLR4-mediated activation of the ERK 1/2, p38 MAPK, and NF-κB signaling pathways (Na et al., 2019l). PM increased TLR4 expression in a lung injury animal model. Lee et al. suggest that the TLR4-MyD88 pathway could be a potential therapeutic target for diesel-induced lung injury (Lee et al., 2020).

Future research will be needed to investigate changes in the permeability of blood and lymphatic vessels. A controlled experimental design in humans will confirm the effects of DEP on lymphangiogenesis and angiogenesis in OM.

## Materials and Methods

### Diesel Exhaust Particles

Standard Reference Material 2975 from the National Institute of Standards and Technology (NIST, Gaithersburg, MD, USA), collected from an industrial forklift truck, was used to generate DEP. DEP preparation was performed following a previously reported protocol (Kim et al., 2016). Briefly, a dry agglomerated DEP powder containing 15 g of a mixed phase (anatase and rutile) was used according to the manufacturer’s instructions. After sonication, the DEP suspension was filtered through a 0.22-µm filter (Millipore, MA, USA).

### HMEEC Culture and Stimulation with DEP

Human middle ear epithelial cells (HMEECs) (kindly provided by Dr. David J. Lim, House Ear Institutes, LA, USA) (Chun et al., 2002) were grown in Dulbecco’s modified Eagle’s medium (DMEM) (Lonza, Walkersville, MD, USA) and bronchial epithelial basal medium (Lonza) (1:1) at 37°C in 5% CO_2_. The medium was replaced every 48 hr until the cells reached 80% confluence. The lipopolysaccharide (LPS) dose was 1 μg/mL, while DEP concentrations of 0, 20, 40, 80, 160, and 320 µg/mL were used. Cell viability measurements were made using a Cell Counting Kit-8 (CCK-8, Dojindo Laboratories, Kumamoto, Japan) according to the manufacturer’s protocol (**Figure 1**). HMEECs were then stimulated in several ways: 1) LPS 1 μg/mL only for 24 hr (LPS); 2) LPS 1 μg/mL for 48 hr and DEP 80 μg/mL for 24 hr (LPS 48 hr + DEP 24 hr); 3) DEP 80 μg/mL for 48 hr and LPS 1 μg/mL for 24 hr (DEP 48 hr + LPS 24 hr) (**Figure 2A**).

### HMEEC Viability Assays

The viability of HMEECs was measured using a CCK-8 (Dojindo Laboratories, Kumamoto, Japan) according to the manufacturer’s instructions (**Figure 1**). The solution was added to each well and incubated for 2 hr at 37°C in 5% CO_2_. The mixture was then shaken at room temperature for 5 minutes. Absorbance at 450 nm was measured with a microplate reader.

### Experimental Design of Animals

Female BALB/c mice aged 6 to 7 weeks were purchased from The Koatech (Pyeongtaek, Korea). The mice were injected with LPS and exposed to 100 µg/m^3^ DEP. BALB/c mice (n = 52) were divided into four groups (n = 13 per group): (1) control, (2) AOM, (3) AOM induction followed by DEP exposure, and (4) DEP exposure followed by AOM induction (**Figure 2B**). Lipopolysaccharide (LPS) extracted from *Pseudomonas aeruginosa* (Sigma Aldrich, St Louis, MO, USA) was dissolved in normal saline and used at a concentration of 1 mg/mL. AOM was induced by a transtympanic injection of a 10 μL emulsion of LPS 1 mg/mL into the middle ear (Tong et al., 2010). The control group received a transtympanic injection of an equal volume of normal saline, and the non-DEP groups were exposed to ambient air through nebulizer idling. The DEP inhalation protocol followed the procedure reported previously (Kim et al., 2016). Briefly, the mice in the DEP inhalation group were exposed to 100 µg/m^3^ DEP for 1 hr a day for 5 days a week in a closed-system chamber attached to an ultrasonic nebulizer (NE-U780, Omron Corporation, Tokyo, Japan). On sacrifice day, otoscopy was performed and measurements were made of the ABR, mRNA levels of lymphangiogenesis-related genes and cytokines, and histology of the middle ear. This study was approved by Seoul National University Hospital Institutional Animal Care and Use Committee [IACUC No. 20-0154-S1A1(1)].

### Transmission Electron Microscopy

The middle ear tissues were fixed with a 4% formaldehyde and 2.5% glutaraldehyde buffer. They were then dehydrated with graded ethanol and embedded in Araldite/Epon. Thin sections were contrasted with uranyl acetate and lead citrate. Observations and photographic records were obtained with transmission electron microscopy (TEM) (JEM-1604; JEOL, Tokyo, Japan) at 80 kV.

### Symptom Score of Acute Otitis Media

To explore the effects of DEP and LPS, otoscopy was used to measure transtympanic structure and effusion 24 hr after the discontinuation of LPS or DEP. Based on the AOM symptom score of effusion and structures measured 24 hr after the last exposure, AOM symptom severity was graded as follows: 1 = normal tympanic membrane; 2 = partial effusion; 3 = effusion without accompanying structural changes of the tympanic membrane; 4 = concomitant structural changes of the tympanic membrane with effusion.

### Auditory Brainstem Response

Audiometry was measured using a SmartEP (Intelligent Hearing Systems, Miami, FL, USA) and high-frequency transducers (HFT9911-20-0035) as previously described (Zheng et al., 1999). All mice were anesthetized through intraperitoneal injection of tiletamine/zolazepam (Zoletil) (Virbac, AH, France) (30 mg/kg) and placed in a small sound-attenuating chamber. The sound stimuli applied were clicks at 4 and 16 kHz. The procedures were similar to those previously described (Park et al., 2020). Mice were tested and monitored until recovery.

### Quantitative Real-Time Polymerase Chain Reaction

Total RNA was extracted using TRIzol (Invitrogen, CA, USA) according to the manufacturer’s instructions. Total RNA was reverse-transcribed into cDNA using the SuperScript III First-Strand Synthesis SuperMix (Invitrogen) at 42°C for 50 minutes and heat-inactivated at 70°C for 15 minutes. qRT-PCR was performed using the Applied Biosystems 7500 (Stratagene, La Jolla, CA, USA) with the QuantiTect SYBR Green PCR kit (Qiagen, Mississauga, ON, Canada) (Moon et al., 2015). The levels of mRNA expression were normalized to the housekeeping gene peptidyl-prolyl cis-trans isomerase A (PPIA). The sequences of primers of IL-1β, TNF-α, TLR4, VEGFA, VEGFC, VEGFR1, VEGF3, and PPIA are listed in **Table 1**.

**Table 1:** The sequence of human and mouse primers in qRT-PCR.

Gene	Forward Sequence (5`-3`)	Reverse Sequence (5`-3`)
*Human*		
PPIA	TCCTGGCATCTTGTCCAT	TGCTGGTCTTGCCATTCCT
TLR4	CCAAGAACCTGGACCTGAGC	TCTGGATGGGGTTTCCTGTC
IL-1β	ACAGATGAAGTGCTCCTTCCA	GTCGGAGATTCGTAGCTGGAT
TNF-α	AGACGCCACATCCCCTGACAA	AGACGGCGATGCGGCTGATG
VEGFA	ACTTCTGGGCTGTTCTCG	TCCTCTTCCTTCTCTTCTTCC
VEGFC	ATGTTTTCCTCGGATGCTGGA	CATTGGCTGGGGAAGAGTTT
VEGFR1	CTTGGATTTTACTGCGGACAG	GGGGACACCATTAGCATGAC
VEGFR3	CTGGACCGAGTTTGTGGAGG	CACATAGAAGTAGATGAGCCG
*Mouse*		
PPIA	GGCAAATGCTGGACCAAA	CATTCCTGGACCCAAAACG
TLR4	TTTATTCAGAGCCGTTGGTG	CAGAGGATTGTCCTCCCATT
IL-1β	TGCCACCTTTTGACAGTGATG	TGGATGCTCTCATCAGGACAG
TNF-α	CTGAACTTCGGGGTGATCGG	GTGGTTTGCTACGACGTGGG
VEGFA	CAGGCTGCTGTAACGATGAA	CTATGTGCTGGCTTTGGTCA
VEGFC	TTTGCCAATCACACTTCCTGC	ACACTGTGGTAATGTTGCTGG
VEGFR1	AAATAAGCACACCACGC	ACCTGCTGTTTTCGATGTTTC
VEGFR3	CCCGAGAGCATCTTTGATAAG	GAAGAGCCTGGAGTCTTAGT

### Enzyme-Linked Immunosorbent Assay

Serum IL-1β and TNF-α levels were quantified using an enzyme-linked immunosorbent assay (ELISA) assay kit (Koma Biotech Inc., Seoul, South Korea) according to the manufacturer’s instructions. Serum extracts were loaded onto the ELISA plate. The absorbance was measured at 450 nm on a microplate reader.

### Immunohistochemical Analysis

Paraffin sections, hematoxylin, eosin, and immunohistochemistry staining methods in tissue were conducted as previously described (Kim et al., 2018). Mice middle ear paraffin block sections were deparaffinized and rehydrated. Non-specific binding was blocked using normal goat serum. We used antibodies for CD45 (1:100, Santa Cruz Biotechnology, Dallas, TX, USA), VEGFA (1:100, Abcam, Cambridge, UK), VEGFC (1:250, Santa Cruz), and CD31 (1:100, Santa Cruz). The following day, the sections were incubated with an ABC kit (Vector Laboratories, Burlingame, CA, USA). The color reaction was developed using a liquid DAB substrate kit (Vector). Stained middle ear tissue data were quantified using ImageJ (National Institutes of Health, Bethesda, MD, USA).

### Statistical Analysis

The data were entered twice into the SPSS statistical software package (ver. 20.0; IBM Corp., Armonk, NY, USA). All data are expressed as means ± standard deviation (SD) or SEM. Group differences were compared using the two-sample t-test, Mann–Whitney test, or the Pearson χ^2^ test for normally distributed, skewed, and categorical data, respectively. One-way ANOVA or two-way ANOVA were used for multiple data. A p-value < 0.05 was considered to indicate statistical significance.

## Data Availability Statement

The original contributions presented in the study are included in the article/**Supplementary Material**. Further inquiries can be directed to the corresponding author.

## Ethics Statement

The animal study was reviewed and approved by Seoul National University Hospital Institutional Animal Care and Use Committee [IACUC No. 20-0154-S1A1(1).

## Author Contributions

All authors were involved in discussing and drafting the article. B-GK, MP designed the experiment and wrote the manuscript. B-GK performed the animal model experiments and analyzed the data. M-GK, DC, B-GK performed the *in vitro* experiments, collected the samples, and assisted in interpreting the data. A-SJ, M-WS, JL, SO assisted in interpreting the data, provided contributions in the conception or design. All authors contributed to the article and approved the submitted version.

## Funding

This research was supported by Basic Science Research Program through the National Research Foundation of Korea (NRF) funded by the Ministry of Education (2019R1I1A1A01061374) and Seoul National University.

## Conflict of Interest

The authors declare that the research was conducted in the absence of any commercial or financial relationships that could be construed as a potential conflict of interest.

## Publisher’s Note

All claims expressed in this article are solely those of the authors and do not necessarily represent those of their affiliated organizations, or those of the publisher, the editors and the reviewers. Any product that may be evaluated in this article, or claim that may be made by its manufacturer, is not guaranteed or endorsed by the publisher.
