# Supplementary figures and images for: Effect of Angiogenesis and Lymphangiogenesis in Diesel Exhaust Particles Inhalation in Mouse Model of LPS Induced Acute Otitis Media
Source: Front Cell Infect Microbiol. 2022 May 11;12:824575. doi: 10.3389/fcimb.2022.824575 (PMC9132252; doi:10.3389/fcimb.2022.824575)

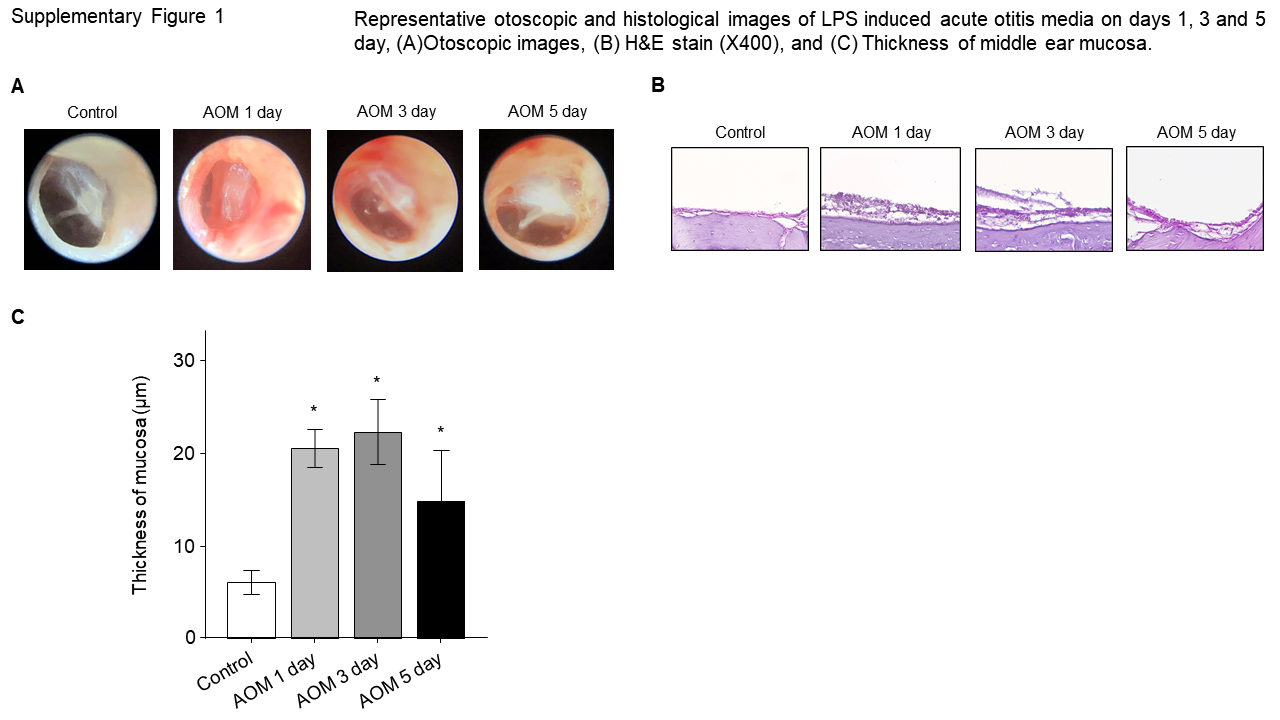

Supplement: Supplementary file 1 [file Image_1.tif]
